# Are Temporal and Tonal Musical Skills Related to Phonological Awareness and Literacy Skills? – Evidence From Two Cross-Sectional Studies With Children From Different Age Groups

**DOI:** 10.3389/fpsyg.2019.00805

**Published:** 2019-04-16

**Authors:** Claudia Steinbrink, Jens Knigge, Gerd Mannhaupt, Stephan Sallat, Anne Werkle

**Affiliations:** ^1^Department of Psychology, University of Erfurt, Erfurt, Germany; ^2^Department of Music, Nord University, Levanger, Norway; ^3^Department of Primary Education and Childhood Research, University of Erfurt, Erfurt, Germany; ^4^Department of Special Needs Education and Rehabilitation, Martin Luther University of Halle-Wittenberg, Halle, Germany; ^5^Department of Special Needs Education and Social Pedagogy, University of Erfurt, Erfurt, Germany

**Keywords:** temporal auditory processing, spectral auditory processing, music processing, phonemic awareness, reading, spelling, rhythm, pitch

## Abstract

Temporal and spectral auditory processing abilities are required for efficient and unimpaired processing of speech and might thus be associated with the development of phonological and literacy skills in children. Indeed, studies with unselected children have found links between these basic auditory processing abilities and the development of phonological awareness, reading, and spelling. Additionally, associations between the processing of temporal or spectral/tonal information in music and phonological awareness/literacy have been reported, but findings concerning relations between music processing and spelling are rather sparse. To gain more insights into the specific, potentially age-dependent relevance of various temporal (e.g., rhythm, tempo) and tonal (e.g., pitch, melody) musical subdomains for phonological awareness and literacy, we adapted five music-processing tasks (three temporal, two tonal) for use with tablet computers and used them in two cross-sectional studies with German children from two age groups: Study 1 was conducted with preschool children (about 5 years of age; without formal reading and spelling instruction) and focused on associations between music processing and phonological awareness. In Study 2, third-graders (about 8 years of age) were investigated concerning relations between music processing, phonological awareness, reading comprehension, and spelling. In both studies, rhythm reproduction and pitch perception turned out to be significant predictors of phonological awareness in stepwise regression analyses. Although various associations between music processing and literacy were found for third-graders in Study 2, after phonological awareness was accounted for, only rhythm reproduction made a unique contribution to literacy skills, namely, to alphabetic spelling skills. Hence, both studies indicate that temporal (i.e., rhythm reproduction) and spectral/tonal (i.e., pitch perception) musical skills are distinctly and uniquely related to phonological awareness in children from different age groups (preschool vs. Grade 3). The finding that rhythm reproduction, an auditory temporal processing skill integrating perceptual and motor aspects of rhythm processing, was especially tightly linked to phonological awareness and literacy corroborates other findings on associations between rhythm processing and literacy development and is of interest from the viewpoint of current theories of developmental dyslexia. The potential relevance of our results for applied research concerning early diagnosis and training of literacy-related skills is discussed.

## Introduction

There is consensus in current research that phonological processing, or the use of phonological information in processing written and oral language (Wagner and Torgesen, 1987), is of special importance for literacy development. One component of phonological processing is phonological awareness, which is defined as one’s awareness of and access to the phonology of one’s language (see Wagner and Torgesen, 1987) and can refer to sound units of different sizes (e.g., syllable, onset and rime, phoneme). In the last decades, empirical research has firmly established that phonological awareness is an important predictor of the development of reading and spelling (e.g., Bradley and Bryant, 1983; Näslund and Schneider, 1996; Caravolas et al., 2012; see Melby-Lervag et al., 2012, for review) and that deficits in phonological awareness are related to developmental dyslexia (e.g., Bruck, 1992; Ramus et al., 2003; White et al., 2006; see Melby-Lervag et al., 2012, for review).

Besides phonological processing abilities, basic auditory processing abilities, such as temporal and spectral auditory processing, might be relevant for the development of phonological abilities as well as the development of reading and spelling. It is assumed that these basic auditory processing abilities might influence reading ability through their effects on children’s ability to extract phonological information from the speech stream (Corriveau et al., 2010). With respect to temporal auditory processing, the ability to process short or rapidly changing auditory information might be a prerequisite for intact phonological development and phoneme perception, as acoustic cues differentiating between phonemes may lie in time windows as short as 20–40 ms (Bishop, 1997; Tallal, 2000). However, intact auditory temporal processing of information in the speech signal that changes more slowly might also be necessary for phonological development, as it allows the segmentation of the speech signal into syllables as well as the accurate perception of speech rhythm and stress patterns (Corriveau et al., 2010). Besides intact temporal auditory processing abilities, intact spectral auditory processing abilities might be necessary for phonological development. Spectral or frequency processing abilities are, for example, required to discriminate between phonemes (e.g., for vowel discrimination in German; see Bohn and Polka, 2001; Steinbrink et al., 2014a), and detection of pitch/frequency changes is important for establishing stress location and for segmenting the speech stream (Ziegler et al., 2012). Indeed, correlational studies with unselected children have shown that both temporal and spectral auditory processing abilities are related to phonological awareness (Corriveau et al., 2010) and that temporal auditory processing abilities are related to reading development (Hood and Conlon, 2004) or the development of reading and spelling (Steinbrink et al., 2014b). Additionally, behavioral studies have reported deficits in temporal auditory processing and/or spectral auditory processing of speech and non-speech stimuli in developmental dyslexia (e.g., Ahissar et al., 2000; Steinbrink et al., 2014a; Christmann et al., 2015; see Hämäläinen et al., 2013 for review). In this context, Ahissar et al. (2000) have proposed that the fine representation of spectral and temporal details of acoustic features facilitates the encoding of acoustic patterns into phonological representations and that in poor readers the salience of representation of phonological parts of speech is degraded by an abnormal representation of inputs in the acoustic stream. Note, however, that the evidence concerning the relevance of basic auditory processing disorders for developmental dyslexia is inconclusive (see Rosen, 2003; Hämäläinen et al., 2013 for review).

Given the potential relevance of temporal and spectral auditory processing abilities for phonological processing and the similarities in processing of music and speech (see McMullen and Saffran, 2004; Patel, 2008), it seems reasonable to assume that music processing abilities might be related to phonological processing, reading, and spelling. A number of correlational studies tested associations between the processing of temporal information in music (e.g., rhythm, meter, tempo) and/or spectral information in music (e.g., harmony, contour, timbre, pitch) and phonological processing and/or reading and spelling abilities: Lamb and Gregory (1993) studied relationships between music perception (pitch and timbre discrimination), phonemic awareness, and reading in children in their first year at school (4–5 years of age). Pitch discrimination, but not timbre discrimination, was related to phonemic awareness and reading. However, Douglas and Willatts (1994), who studied associations between music perception (pitch and rhythm discrimination) and reading and spelling abilities in children aged 7–8 years, found that, after partialing out vocabulary, only rhythm, but not pitch was related to reading and spelling. Anvari et al. (2002) used a set of musical tasks (rhythm discrimination and reproduction, melody discrimination, chord discrimination, and chord analysis) to study relations between music processing, phonemic awareness, and reading in 4- and 5-year-old children. Factor analyses revealed a single global music factor including all five music tasks for the 4-year-old children and two separate music factors (one that corresponded to pitch perception and included the melody discrimination task and the two chord tasks; another that corresponded to rhythm perception and included the two rhythm tasks) for the 5-year-old children. In 4-year-old children, music processing was related to phonemic awareness and reading and uniquely predicted reading (over and above the contribution of phonemic awareness). In 5-year-old children, both pitch and rhythm processing were related to phonemic awareness, but only pitch processing, not rhythm processing, was related to reading and made a unique contribution to its prediction. Loui et al. (2011), using a combined pitch perception-reproduction-measure, found a significant relation between pitch processing and phonemic awareness in children between 7 and 9 years of age. In the context of a training study, Rautenberg (2015) found relations between music perception abilities and reading in German first-graders (mean age 7 years). Tonal music perception abilities and rhythmic music perception abilities were both measured with two tasks each. Rhythmic music perception ability at pretest was related to reading accuracy, but tonal music perception ability was not. Finally, Degé et al. (2015) tested associations between musical abilities (various music perception and reproduction tasks) and phonological awareness (among other phonological processing abilities) in preschool children. Both music perception (pitch perception, rhythm perception, tone length perception) and music reproduction (singing a song, rhythm reproduction) abilities were related to phonological awareness. With respect to the relevance of deficits in music processing for developmental dyslexia, studies support the view that musical timing skills (rhythm, meter, rapid temporal processing) might be impaired in dyslexia while results concerning deficits in the processing of spectral aspects of music (pitch, melody) are less consistent (Overy, 2003; Overy et al., 2003; Forgeard et al., 2008; Huss et al., 2011).

Taken together, the results of various correlational studies with unselected children point to relations between music processing and phonological awareness/literacy skills. In the current context, we view these relations from the perspective of potential relations between basic auditory processing abilities (temporal auditory processing, spectral auditory processing) and phonological awareness/literacy. Thus, in the following, we will summarize these results with respect to (a) musical abilities concerned with the processing of temporal information in music (e.g., rhythm perception and reproduction, tone length perception, meter perception), which are termed *temporal musical skills*, and (b) musical abilities concerned with the processing of spectral/frequency information in music (e.g., pitch perception, tonal music perception, melody and chord perception), which are termed *tonal skills*.

Studies relating music processing skills to phonological awareness rather consistently suggest that both temporal and tonal musical skills are associated with phonological awareness (Lamb and Gregory, 1993; Anvari et al., 2002; Loui et al., 2011; Degé et al., 2015). However, these abilities in children develop from phonological awareness with respect to larger phonological units, such as syllables and rhymes, to phonological awareness with respect to smaller phonological units, i.e., phonemes (Goswami and Bryant, 1990; Skowronek and Marx, 1993; Carroll et al., 2003). Phonological awareness for larger phonological units might be related to different aspects of music processing than phonological awareness for phonemes (phonemic awareness). Moreover, many children seem to develop phonemic awareness only in the context of learning an alphabetic writing system (Wimmer et al., 1991). Thus, for children who have not yet received formal reading instruction, associations between music processing and phonological awareness for larger phonological units might be more relevant while for children receiving reading instruction, phonemic awareness might be of greater interest. Indeed, Degé et al. (2015) found in their study with German preschool children cited above (note: in Germany, children do not receive formal reading instruction before school entry) that phonological awareness for larger phonological units was involved in more associations with music perception and reproduction than phonemic awareness was. Studies investigating relations between music processing abilities, phonological awareness, and reading have usually used a mixture of phonological awareness tasks focusing on phonological units of different sizes, which were analyzed together (Lamb and Gregory, 1993; Anvari et al., 2002; Loui et al., 2011). Thus, they cannot inform us as to how relations between phonological awareness and music processing might depend on the size of the phonological unit under consideration. The two studies reported in this paper were also conducted in Germany. The same set of music processing tasks (including tasks testing temporal musical skills and tasks testing tonal skills) was used both with German preschool children in their last kindergarten year (Study 1) and with German primary school children, who had just started Grade 3 (Study 2). Additionally, both groups were tested with phonological awareness tasks. For preschool children, these tasks consisted of phonological awareness tasks focusing on larger phonological units and phonemic awareness tasks. These types of tasks were analyzed separately, allowing us to investigate whether phonological awareness with respect to phonological units of different sizes might be associated with different types of temporal and tonal musical skills. The third-graders were tested with respect to phonemic awareness skills only, as phonological awareness tasks focusing on larger phonological units are not appropriate for children of that age/reading experience (Mannhaupt, 2001).

Concerning the question whether temporal and tonal musical skills are related to reading, the available results are rather inconsistent: While Anvari et al. (2002) found that in 5-year-old-children, tonal skills were related to reading, but temporal musical skills were not, two other studies with older children (7–8 years of age) came to the opposite conclusion (Douglas and Willatts, 1994; Rautenberg, 2015). These divergent results might indicate that the relations between specific aspects of music processing (temporal musical skills vs. tonal skills) and reading change with age or reading experience. Based on these results, in our second study (third-graders, mean age 8 years), which investigated relations between music processing abilities and reading, we expected stronger relations between temporal musical skills and reading than between tonal skills and reading. When considering potential differences between the three studies mentioned above that might explain the diverging results, it should, however, also be mentioned that the study by Anvari et al. (2002) is the only study that included a reproduction measure, while the results of the other two studies exclusively relied on perception measures: Anvari et al. (2002) measured both rhythm discrimination and rhythm reproduction and used a combined rhythm processing measure in their analyses, as both tasks loaded on the same factor. This approach made perfect sense in the context of their study. However, a separate analysis of rhythm perception vs. rhythm reproduction abilities would enhance our understanding of the specific role of perceptual vs. motor aspects in potential relations between rhythmic abilities and reading (see also Overy et al., 2003). In the studies reported here, rhythm perception and rhythm reproduction were measured and analyzed separately.

Finally, most correlational studies investigating relations between music processing and literacy skills (with the exception of Douglas and Willatts, 1994) have focused on associations between music processing and reading, while information concerning relations between music processing and spelling is sparse. However, current models of literacy development postulate that reading and spelling develop jointly; in different phases of literacy development, reading acts as a pacemaker for spelling and vice versa (Frith, 1986). Thus, it is of interest to learn more about potential associations between music processing abilities and spelling. In Study 2 (third-graders), various spelling strategies were measured (alphabetic, orthographic, morphological). We expected that music processing abilities as well as phonemic awareness skills would be especially related to alphabetic spelling strategies as these are based on phoneme–grapheme mappings.

Taken together, the two studies reported here aim to extend our understanding of relations between music processing skills, phonological awareness, and literacy by

– using a battery of music processing tasks spanning temporal and tonal musical skills, thus allowing us to consider both the ability to process temporal and spectral information in music (Study 1 and Study 2);
– measuring and analyzing rhythm perception and reproduction skills separately to disentangle the relevance of perceptual aspects of rhythm processing from those integrating perceptual and motor aspects of rhythm processing (Study 1 and Study 2);
– using exactly the same set of music processing tasks in two different age groups (Study 1: preschool children; Study 2: third-graders), which allowed us to look at potential differences in relations between music processing and phonological awareness that might depend on the size of the phonological units under consideration;
– relating music processing abilities not only to reading but also to spelling (Study 2).

## General Methods

### Music Tasks

For the purpose of the current research, music processing tasks measuring tonal and temporal musical skills were required that were appropriate for use with children within a relatively broad age range (from about 5 years of age, the age of preschoolers in Germany, to about 8–9 years of age, the age of third-graders in Germany). An extensive literature review yielded two established paper-and-pencil-tests containing appropriate tasks (literature review and detailed discussion of selected instruments in Knigge et al., unpublished): The *Musikscreening für Kinder I* (*Music Screening for Children I*; Jungbluth and Hafen, unpublished) was developed for the age range 5;0 to 8;6 (years;months) and measures various musical abilities (melodic reproduction, rhythm reproduction, meter reproduction, melody perception, pitch perception, rhythm perception, tone length perception, and tempo perception). The *Montreal Battery of Evaluation of Musical Abilities* (MBEMA; Peretz et al., 2013), a “tool for evaluating children’s musical abilities across age and culture,” was successfully used by Peretz et al. (2013) with Chinese and Canadian children aged 6–8 years. The battery consists of five tests, measuring contour, interval, scale, and rhythm perception as well as recognition memory.

From these two instruments, 10 tasks measuring the processing of temporal (e.g., rhythm) or spectral information (e.g., pitch) in music were chosen. To facilitate stimulus presentation as well as data entry/analysis and to allow for group testing, we adapted these tasks for use with a tablet computer. Thus, we aimed to enhance the objectivity, reliability, and validity of the assessment (for a detailed discussion of potential advantages and psychometric effects of computer-based assessment, see Jurecka, 2008; Buerger et al., 2016; for a music-specific discussion, see Hasselhorn and Knigge, in press). As the adaptation to computer-based testing required changes in the instructional and presentation format, and to assure that each task was appropriate for the broad age range intended (preschool to Grade 3, that is, for ages 5–8), we conducted extensive piloting including an evaluation of the psychometric quality of the tasks. The tasks were piloted with preschool children as well as primary school children from Grades 1–3. As a result of these piloting studies (in the course of which items with difficulty indices outside 90 > *P_i_* > 10 or item discrimination below 0.20 were removed), five tasks were retained for use in further studies (the decision which tasks to select was based on theoretical considerations, ease of conducting the computerized version of the tasks, and the psychometric quality of the adapted tasks). Of these five tasks, three tasks measured temporal musical skills (rhythm reproduction and tempo perception, adapted from the *Music Screening for Children I*; rhythm perception, adapted from the MBEMA) and two tasks measured tonal skills (pitch perception, adapted from the *Music Screening for Children I*; contour perception, adapted from the MBEMA). The item set of each task was reduced by a selection procedure based on psychometric analyses with data from four data sets (data from a pilot study with preschool children, a pilot study with children from Grades 1–3, and the two studies presented here; *N* = 282). The selected items yielded acceptable to very good reliabilities of the tasks (Cronbach’s alpha between 0.61 and 0.90). Thus, accuracy of measurement was as high as possible and the duration of testing as short as possible (to accommodate the needs of young children).

### General Information Regarding Music Assessment Procedure

Based on considerations concerning the appropriate and required testing procedure for younger vs. older children, in Study 1 (preschool children; about 5 years of age), single participants were tested while in Study 2 (Grade 3; about 8 years of age), the participants were tested in small groups of up to 9 children. A trained research assistant conducted the music tasks with the participating child (Study 1) or children (Study 2). Apart from this and a difference in the repetition of practice items described below, the procedure was the same in both studies.

All tasks were adapted for use with iPads and headphones. The music stimuli, the feedback for the example items, and the possibility to respond were implemented in an iPad app. The research assistant controlled the app via a Wi-Fi control server to start each task, each item example, and the test item sequence.

In the instruction phase, a general introduction was given verbally by the research assistant. The specific tasks and corresponding response options were either explained completely by the research assistant (contour and rhythm tasks) or by the assistant in conjunction with a short instruction-video played on the iPads (pitch and tempo tasks). Subsequent to these explanations, practice items were presented. After each practice item, the child received feedback via an image on the screen (thumbs up in green for correct responses and thumbs down in red for false responses). If the music tasks were conducted with a single participant (Study 1), practice items were repeated whenever the child gave an incorrect answer. If the music tasks were conducted with small groups of children (Study 2), each practice item always was repeated once after the research assistant had explicitly indicated the correct response option to give those children in the group who had given an incorrect answer the opportunity for correction.

During the test item sequence, each child responded individually and worked at his/her individual pace. After the child had given a response, the presentation of the following test item started immediately (without any delay) or, alternatively, 5 s after the end of the stimulus presentation (in case no response occurred). No feedback was given during testing. All responses were transferred immediately to the control server via Wi-Fi and saved to a database file. At the end of each task, the app presented a “STOP” sign. When all participating children had finished the current task, the instructor selected the next task for all children via the server and commenced the instruction phase for this task.

At the beginning of a session, the prepared tablets and headphones lay on the desk, one for each child. The children were asked to put on their headphones and push a button in the app, which started a sound example. This way the children could check if the headphones were working and if the loudness was appropriate.

In the following, the five tasks will be described in the order in which they were presented in both studies, and their reliability will be reported (detailed information regarding our adaption procedure and psychometric evaluation is to be published in Knigge et al., unpublished).

#### Pitch Perception

This task from the *Music Screening for Children I* assesses children’s perception of pitch differences. Children are asked to compare two tones played with a computer-generated organ sound (each tone has a length of 1750 ms, with a 750 ms pause in between) and to decide whether the two tones are identical, the second tone is higher than the first or the second tone is lower than the first. The metaphor of an elevator either not moving (two identical tones), going up (second tone higher) or going down (second tone lower) is used to introduce the task and response options. If the two tones are identical, the children have to select a picture of a horizontal line; if the second tone is higher, then the children have to select a picture of an upward-pointing arrow; if the second tone is lower, the children have to select a picture of a downward-pointing arrow. Pitch differences vary between the intervals minor third and major sixth: only chromatic notes between C#_3_ and F#_4_ are used (see Jungbluth and Hafen, unpublished).

In our computer-assisted version of the task, the pictures of a horizontal line, an upward- and a downward-pointing arrow were implemented as response buttons on the iPad. As the pitch perception task was the first task in the test scenario, children received five item examples to get used to the general procedure (responding via iPads, use of buttons, etc.) and to practice the specific task. After the last example, the testing sequence was initiated by the research assistant.

The original pitch perception task by Jungbluth and Hafen, unpublished consists of 10 items. In our study, first piloting and psychometric analyses had already led to the exclusion of two items so that eight items were used in the four studies on which the combined psychometric analyses were based. These items yielded good reliability. The internal consistency (Cronbach’s alpha) of the task was *α* = 0.787.

#### Contour Perception

This task from the *MBEMA* measures the perception of contour-modification of a short melody (5–9 tones long). The melodies are computer-generated and played with ten different sounds (e.g., piano, marimba, guitar, flute), whereby the sounds change after every item, but not between the target and comparison melodies. By using the same-different paradigm, children are asked to compare two melodies that are either the same or have one modified note (the contour-violating modification occurs only regarding the pitch of this single note; rhythmic structure, key, and tempo are always the same between target and comparison melody). The first and last note of a melody always remain unchanged; all other notes are modified across melodies (see Peretz et al., 2013 for details).

In the iPad-app, the response button used for representing a difference between the target melody and comparison melody consisted of a green triangle and a red circle. The response button for identical melodies showed two green triangles. Two item examples were used for practicing. After the second example, the testing sequence was initiated by the research assistant.

The original task consists of 20 items (see Peretz et al., 2013). In our study, first piloting led to the removal of seven items from the item set (due to unsatisfying item discrimination and/or difficulty indices). Of the 13 items used for psychometric analysis in the four studies, eight yielded acceptable reliability measures. The internal consistency of this task was *α* = 0.666.

#### Tempo Perception

For the assessment of tempo perception with the *Music Screening for Children I*, the child hears a target sequence and a comparison sequence each with five beats played with a claves sound in the same or different tempo (tempo varies between 84 and 120 bpm; minimum difference between target and comparison sequence is 8 bpm, maximum difference is 20 bpm). The child responds by choosing one out of three response options: (a) the first sequence was faster than the second one; (b) the second sequence was faster than the first one; and (c) both sequences had the same tempo. Each of the two sequences of five beats is visualized as a line of five small circles: the faster alternative is visualized with short distances between the circles, the slower alternative with longer distances, and the equally fast sequences with identical distances between the five circles of the two lines (see Jungbluth and Hafen, unpublished).

In the computer-assisted adaptation of the task, the three response options were implemented as response buttons on the tablet. The pictures representing the response options were more or less the same as those in the original task, but squares were used instead of circles. After two item examples, the testing sequence was initiated by the research assistant.

The original task consists of 10 items (see Jungbluth and Hafen, unpublished). In our study, prior piloting and psychometric analyses had already led to the exclusion of one item, so that nine items were used in the four studies on which the combined psychometric analyses were based. Due to these analyses, four additional items were excluded from the item set as their item discrimination indices were too low. The internal consistency of the remaining five items was *α* = 0.612.

#### Rhythm Perception

In the *MBEMA*, the assessment of rhythm perception uses the same melodies as the contour perception task and, once again, the same-different paradigm (see above). In the rhythm perception task, the comparison melody is changed regarding the durations of two consecutive notes. The number of notes and original meter stays unchanged between target and comparison melody while the rhythmic grouping of notes is modified (either two quarter notes are changed to a dotted quarter and an eighth note or the order of two successive duration values is reversed; see Peretz et al., 2013 for details).

Using the iPad app in our study, the child responded via the same response buttons as in the contour task (see above). After two item examples, the testing sequence was initiated by the research assistant.

The original task consists of 20 items (see Peretz et al., 2013). After first piloting in our study, 12 items remained in the item set, which were used in the psychometric analysis. All items yielded acceptable reliability measures. The internal consistency of the task was *α* = 0.694.

#### Rhythm Reproduction

In the original rhythm reproduction task from the *Music Screening for Children I*, the child hears a rhythm pattern played on a keyboard (tone A_4_) consisting of four to eight tones (quarter, eighth, and semi quaver notes as well as dotted eight notes and triplets). The pattern always has the same tempo and the same duration of one bar in a four-quarter beat. The child is requested to repeat the rhythm pattern on the keyboard using the same key (A_4_) (see Jungbluth and Hafen, unpublished).

In our iPad version of the task, the rhythms were presented via the tablet using a claves sound. The child’s task was to repeat the rhythmic pattern by tapping on a large blue and round button on the tablet screen. Tapping on this button produced a tapping sound, which was the same as the target pattern sound (=claves). To familiarize children with the procedure of reproducing rhythms via tapping on the tablet, the research assistant gave each of them three examples, which she/he repeated once while encouraging the children to do their best to reproduce the rhythm even more exactly than before. After the third example, the testing sequence was initiated by the research assistant. After hearing the target rhythm, the child tried to reproduce the rhythm and subsequently pushed a check mark button to proceed to the next test item.

The original task consists of 10 items (see Jungbluth and Hafen, unpublished). The quality of the rhythm reproduction per item in our study was categorized on a scale from 0 to 4 points (0 = rhythm not recognizable, far too few/too many tones, tempo unstable; 1 = only a part of the rhythm *or* the amount of tones are correct, *or* the tempo is stable; 2 = stable tempo *and* part of the rhythm is correct; 3 = rhythm *and* tempo correct in general, but with small inaccuracy; 4 = perfect solution), resulting in a maximum score of 40. The task was scored by two research assistants with sufficient musical expertise who were trained using responses produced by children from an independent data set by the second author, a professor of music education. The training period continued as long as necessary until consistency between trainer and trainee was high enough. The scoring itself was executed in the way that the research assistants always heard the original first and then scored the child’s response against the original.

All items were retained after piloting and psychometric analysis, yielding an internal consistency of *α* = 0.902.

## Study 1 and Study 2: Methods and Results

### Study 1: Music Processing and Phonological and Phonemic Awareness in German Preschool Children

#### Participants

Four day-care centers in the city of Erfurt (Thuringia, Germany) participated in the study. The study was conducted with children in their last kindergarten year (10 months before school entry). Altogether, parents from 59 children returned a signed written informed consent in accordance with the Declaration of Helsinki to allow the participation of their child (for details concerning ethical approval, see Study 2; both studies were equivalent in terms of the general procedure, but Study 2 contained the measurement of additional constructs that were not included in Study 1). Of these children, five had to be excluded because they were not native speakers of German, they were not able to participate in both sessions of the study, or their data from the music tasks were lost due to technical failure. Thus, the sample of the current study consisted of 54 children (30 male, 24 female) on which all further analyses were based. The mean age of the participants was 5;9 (years;months; range: 5;1 to 6;9).

#### Measures

Data were collected in November and December 2017 during the morning in a quiet room at the children’s day-care centers. During testing sessions, children had the option to terminate testing at any time.

In the first session, the five music tasks were conducted as described in detail above in the General Methods section (duration of the session: 25–35 min). For the analysis of the four perceptual tasks (pitch perception, contour perception, tempo perception, and rhythm perception), raw scores were used. For the analysis of rhythm reproduction abilities, results per item were scored on a scale from 0 to 4 as described above. The data set of this task contains missing data for six children who pressed the “Continue”-button in a restricted number of items too early, i.e., before actually performing the tapping task (five children: one item; one child: two items). For these six children, the average individual score of the executed items (nine items for five children and eight items for one child) was used to estimate the scores of the missing items. Ten percent of the rhythm reproduction items were coded by two individual raters. Intra-class correlations (ICCs) were used to calculate inter-rater reliability, which yielded satisfying ICCs (0.96 > ICC > 0.71).

In the second session, which was performed on another day (usually about 2–3 days after the first session) and lasted about 15–25 min, children’s phonological processing abilities (phonological awareness, phonological short-term and working memory, naming speed) were measured using the *Würzburger Vorschultest – Modul Schriftsprachliche Vorläuferfertigkeiten* (WVT; Endlich et al., 2016). In the current context, we will concentrate on the phonological awareness scale of this diagnostic test, consisting of five subscales. Of these five subscales, two are concerned with phonological awareness abilities addressing larger phonological units (detection of a syllabic vowel onset, rhyming). These subscales are termed *phonological awareness* scales in the following. The other three subscales are concerned with phonological abilities on a phonemic level (detection of a consonantal onset, phoneme synthesis, phoneme analysis) and are thus termed as *phonemic awareness* scales in the following. The *WVT* is not age-normed but normed with respect to the number of months left before the child starts school (the test can be applied 10 or 4 months before school entry). The test does not provide separate T-scores for phonological vs. phonemic awareness. Therefore, as data from all children were collected about 10 months before school entry, we used raw scores in our analyses.

As children in Germany do not receive formal reading and spelling instruction before school entry, it was not possible to measure participants’ early reading and spelling skills. We did not assess whether the participating children receive any form of systematic reading instruction at home. Studies document, however, that literacy skills in German preschool children are indeed quite limited: Näslund and Schneider (1996) found that about 3 months before school entry, 46% of the children could name between zero and two letters (of a total of 26 letters, presented in random order) in a letter recognition task. In a recent study by Schmitterer and Schroeder (2019), preschool children received a letter recognition task, in which the correct letter out of two response alternatives had to be identified. With this procedure, children identified on average about 24 (10 months before school entry) and about 27 (4 months before school entry) letters correctly (total number of trials = 32; but note that a score of 16 can be achieved by guessing). Schmitterer and Schroeder (2019) additionally measured early reading skills 4 months before school entry and 2 months after school entry with two word-picture-matching tasks. The results indicated that both shortly before and shortly after school entry children were not able to read.

#### Results

##### Correlational analysis

Table 1 summarizes the descriptive statistics of the preschool sample. The results of a correlational analysis including all measures presented in this table are shown in Table 2.

**Table 1:** Descriptive statistics of the preschool sample (*N* = 54).

Measure	*M*	*SD*
Age in months	69.52	4.17
Phonological awareness (raw score, max. = 16)	10.19	3.46
Phonemic awareness (raw score, max. = 24)	7.30	4.08
Pitch perception (raw score, max. = 8)	2.78	1.62
Contour perception (raw score, max. = 8)	4.48	2.09
Tempo perception (raw score, max. = 5)	1.54	1.21
Rhythm perception (raw score, max. = 12)	6.70	2.20
Rhythm reproduction (response to each item categorized on a scale from 0 to 4; max. = 40)	16.77	10.12

**Table 2:** Intercorrelations between measures in preschool children (*N* = 54).

	1	2	3	4	5	6	7	8
1. Age (months)	–							
2. Phonological awareness	–0.06	–						
3. Phonemic awareness	0.19	0.39**	–					
4. Pitch perception	0.06	0.33*	0.13	–				
5. Contour perception	0.12	0.27*	0.26	–0.01	–			
6. Tempo perception	0.16	0.22	0.24	–0.01	0.14	–		
7. Rhythm perception	0.41**	0.30*	0.35*	0.21	0.28*	0.26	–	
8. Rhythm reproduction	0.08	0.43**	0.35**	0.04	0.10	0.28*	0.30*	–

*^∗^*p* < 0.05, ^∗∗^*p* < 0.01.*

As can be seen from Table 2, age was significantly related to rhythm perception in the preschool sample. Thus, age was included as a predictor in the regression analyses presented below. As was expected, phonological and phonemic awareness were significantly related to each other. The two music tasks measuring tonal skills (pitch perception and contour perception) were not associated with each other, but two of the three correlations between the temporal musical skills were significant. Additionally, contour perception and rhythm perception were significantly related.

Phonological awareness was significantly related to the two tonal musical tasks (pitch perception and contour perception) as well as to the two rhythm processing tasks. These rhythm processing tasks were also the only music processing tasks that were significantly associated with phonemic awareness.

##### Regression analyses

Using stepwise regression procedures, we evaluated the relevance of music processing abilities for the prediction of phonological and phonemic awareness. Age was entered first as a control variable, followed by the five music processing tasks in the second block. Two models were significant for the prediction of phonological awareness: in Model 1, explaining 17.2% of the variance in phonological awareness (adjusted *R*^2^), phonological awareness was significantly predicted by rhythm reproduction, *t*(53) = 3.41, *p* < 0.01; standardized *β* = 0.43. In Model 2, a combination of rhythm reproduction, *t*(53) = 3.53, *p* < 0.01; standardized *β* = 0.43, and pitch perception, *t*(53) = 2.66, *p* < 0.05; standardized *β* = 0.32, explained 26.2% of the variance in phonological awareness. Phonemic awareness was significantly predicted by rhythm reproduction, *t*(53) = 2.70, *p* < 0.05; standardized *β* = 0.36, explaining 11.0% of the variance. In none of the analyses was age a significant predictor.

### Study 2: Music Processing, Phonemic Awareness, and Literacy Skills in German Third-Graders

#### Participants

Third-graders from six schools in and around the city of Erfurt (Thuringia, Germany) participated in the study. Altogether, 121 children participated. Of these, 25 children had to be excluded from data analysis as they were not native speakers of German or were not able to participate in all tasks or sessions. Thus, the final sample of the current study, on which all further analyses were based, consisted of 96 children (42 male, 54 female) in the age range from 7;10 to 10;5 (mean age: 8;9).

The study was carried out in accordance with relevant Thuringian laws (Thuringian school law, Thuringian data protection law) and followed the recommendations of the Thuringian Ministry of Education, Youth, and Sports. Following the guidelines for conducting empirical studies in Thuringian schools, the protocol was approved by the local school authorities (Staatliches Schulamt Mittelthüringen, Weimar, Thuringia). Approval by an ethics committee was not required as per applicable institutional and federal (Thuringian) guidelines and regulations. The children’s parents gave written informed consent in accordance with the Declaration of Helsinki. During testing sessions, children had the option to terminate testing at any time.

#### Measures

All measurements were undertaken in the morning in the course of regular school days in a quiet room at the children’s schools. Data of all children were collected within two sessions shortly after the start of the school year (September 2017). In the first session (a group session in which all participating children from a particular class were tested; duration: one school lesson, i.e., up to 45 min), reading and spelling abilities were assessed. In the second session (in which children from a particular class were split into smaller groups of up to nine participating children; duration: two school lessons, i.e., up to 90 min, including a break), music abilities and phonemic awareness were measured.

Reading and spelling abilities were measured with standardized and normed German reading and spelling tests. As data were collected at the beginning of Grade 3, norms from the end of Grade 2 were used. The reading test *Ein Leseverständnistest für Erst- bis Sechstklässler* (ELFE 1–6; Lenhard and Schneider, 2006) is a speed test measuring reading comprehension via silent reading in children from Grades 1–6 on three scales, assessing word comprehension, sentence comprehension, and text comprehension. The paper-and-pencil-version of the test was used. Due to time constraints, only two of the three scales, word and sentence comprehension, were assessed. Raw scores per scale were transformed into *T* values. A principal component analysis using the T-scores of the two scales revealed that the two scales of the *ELFE 1-6* loaded on a single factor, accounting for 83.86% of the variance, with equal factor weights for both scales. Thus, in all further analyses, a single reading comprehension variable consisting of the average T-score from the two scales measured was used.

Spelling abilities were assessed with the *Hamburger Schreibprobe 2* (HSP 2; May, 2012). The *HSP 2* measures spelling abilities via the writing of single words and sentences and can be analyzed with respect to the number of correctly written graphemes (max. 148 correctly spelled graphemes) as well as the usage of three writing strategies (alphabetic strategy, orthographic strategy, morphological strategy). The analysis of writing strategies is performed by focusing on the spelling of specific parts of words that is indicative of the use of an alphabetic (maximum score: 20), orthographic (maximum score: 15), or morphological (maximum score: 10) writing strategy. *T* values for all four measures were the basis of the analyses performed.

Music abilities were assessed with five scales from the *Musikscreening für Kinder I* (*Music Screening for Children I*; Jungbluth and Hafen, unpublished) and the MBEMA (Peretz et al., 2013), as described in detail in Section “General Methods.” Raw scores per scale were used in the four perceptual tasks (pitch perception, contour perception, tempo perception, rhythm perception). In the rhythm reproduction task, children’s answers were categorized with respect to a scale ranging from 0 to 4 points, as described above, yielding a maximum score of 40.

Phonemic awareness was measured with the *Kaiserslauterer Gruppentest für Lautbewusstheit* (*KaLaube*; Klatte et al., unpublished). The KaLaube is a speed test and was conceptualized as a group test (phonemic awareness tasks are performed with the help of pictures of objects, e.g., a picture of a ladder [*Leiter*, German] = >does this word contain the phoneme /l/?) for use with children from Grades 1–3. The *KaLaube* consists of three scales measuring phoneme identification, phoneme elision, and phoneme substitution. Current evaluations of the psychometric quality of the *KaLaube* demonstrate satisfactory reliability and validity of the test (Bergström et al., 2017). However, the *KaLaube* has not yet been normed. In the context of the current study, all three scales were used to assess phonemic awareness. A principal component analysis using the z-standardized raw scores of the three scales revealed that all three scales of the *KaLaube* loaded on a single factor, accounting for 51.07% of the variance. Thus, in all further analyses, a single phonemic awareness variable consisting of the factor-weighted average z-score derived from the z-standardized raw scores of the three scales was used.

#### Results

##### Correlational analysis

Table 3 presents the descriptive statistics for all variables measured. Bivariate correlations between these measures are shown in Table 4.

**Table 3:** Descriptive statistics of the Grade 3 sample (*N* = 96).

Measure	*M*	*SD*
Age in months	105.23	5.46
Reading comprehension (mean T-score)	51.67	9.69
Number of correctly spelled graphemes (T-score)	52.32	10.41
Alphabetic spelling strategy (T-score)	52.22	8.79
Orthographic spelling strategy (T-score)	53.48	10.26
Morphological spelling strategy (T-score)	53.15	8.84
Phonological awareness (mean weighted z-score)	0.03	0.49
Pitch perception (raw score, max. = 8)	5.71	2.24
Contour perception (raw score, max. = 8)	6.61	1.28
Tempo perception (raw score, max. = 5)	2.38	1.52
Rhythm perception (raw score, max. = 12)	9.36	1.91
Rhythm reproduction (response to each item categorized on a scale from 0 to 4; max. = 40)	27.20	7.78

**Table 4:** Intercorrelations between measures in Grade 3 children (*N* = 96).

	1	2	3	4	5	6	7	8	9	10	11	12
1. Age (months)	–											
2. Reading comprehension	–0.12	–										
3. Number of correctly spelled graphemes	–0.15	0.70**	–									
4. Alphabetic spelling strategy	–0.18	0.53**	0.78**	–								
5. Orthographic spelling strategy	–0.07	0.68**	0.86**	0.59**	–							
6. Morphological spelling strategy	0.03	0.70**	0.79**	0.50**	0.75**	–						
7. Phonemic awareness	–0.18	0.46**	0.49**	0.43**	0.39**	0.30**	–					
8. Pitch perception	0.03	0.20	0.30**	0.38**	0.26**	0.20*	0.37**	–				
9. Contour perception	–0.10	0.03	0.12	0.10	0.09	–0.04	0.14	0.14	–			
10. Tempo perception	–0.09	0.21*	0.15	0.02	0.18	0.20*	0.12	0.20	0.10	–		
11. Rhythm perception	–0.16	–0.00	0.19	0.21*	0.10	0.09	0.24*	0.14	0.40**	0.21*	–	
12. Rhythm reproduction	–0.07	0.14	0.38**	0.44**	0.25*	0.13	0.38**	0.40**	0.31**	0.17	0.54**	–

*^∗^*p* < 0.05, ^∗∗^*p* < 0.01.*

As can be seen from the correlation matrix in Table 4, children’s age was not related to any of the other measures. Thus, age was not included as a predictor variable in the regression analyses presented below. As was expected, reading and spelling measures were strongly related to each other (all *p*-values < 0.01). Additionally, reading and spelling abilities were associated with phonemic awareness abilities (all *p*-values < 0.01).

Some, but not all, of the music processing abilities were significantly correlated, indicating that the five music measures tap different aspects of music processing abilities. Results of the two tasks measuring tonal skills (pitch perception and contour perception) were not related; however, there were relations between most of the three tasks capturing temporal musical skills (tempo perception, rhythm perception, rhythm reproduction). Three of the significant correlations (pitch perception – rhythm reproduction; contour perception – rhythm perception; contour perception – rhythm reproduction) suggest that the ability to process temporal vs. spectral aspects of music is related in third-grade children.

In terms of relations between music processing abilities and phonemic awareness/literacy measures, the correlation analysis showed that pitch perception, rhythm perception, and rhythm reproduction were related to phonemic awareness. Only one significant relation (tempo perception – reading comprehension) was found between music processing measures and reading comprehension. However, music processing abilities showed various associations with spelling abilities, especially pitch perception and rhythm reproduction were related to spelling.

##### Stepwise regression analyses

A regression-analytic approach was taken to investigate whether music processing abilities predict phonemic awareness and literacy. In the first step, to explore the amount of variance explained by music processing abilities, we computed stepwise regression analyses using only the five music processing tasks, entered simultaneously, as predictors of phonemic awareness and literacy skills. In the second step, to learn something about the unique contribution of music processing abilities to reading and spelling skills, phonemic awareness was entered into the analysis first, before the contribution of music processing measures was accounted for.

###### Relations between music processing abilities, phonemic awareness, and literacy skills in third-grade children

Using a stepwise regression procedure, we used the five music processing measures to predict phonemic awareness as well as reading and spelling skills. For phonemic awareness, two regression models were significant. In Model 1, rhythm reproduction significantly predicted phonemic awareness, *t*(95) = 3.94, *p* < 0.001; standardized *β* = 0.38. With this model, rhythm reproduction explained 13.2% of the variance (adjusted *R*^2^) in phonemic awareness. In Model 2, a combination of rhythm reproduction, *t*(95) = 2.69, *p* < 0.01; standardized *β* = 0.27, and pitch perception, *t*(95) = 2.55, *p* < 0.05; standardized *β* = 0.26, explained 18.1% of the variance in phonemic awareness.

Reading comprehension was significantly predicted by tempo perception, *t*(95) = 2.10, *p* < 0.05; standardized *β* = 0.21, which explained 3.5% of the variance. The only significant predictor of the number of correctly spelled graphemes was rhythm reproduction, explaining 13.3% of the variance, *t*(95) = 3.95, *p* < 0.001; standardized *β* = 0.38. In terms of the prediction of an alphabetic spelling strategy, two regression models were significant: in Model 1, 18.6% of the variance in alphabetic spelling was explained by rhythm reproduction, *t*(95) = 4.76, *p* < 0.001; standardized *β* = 0.44. In Model 2, a combination of rhythm reproduction and pitch perception explained 22.7% of the variance, *t*(95) = 3.49, *p* < 0.01; standardized *β* = 0.34 and t(95) = 2.46, *p* < 0.05; standardized *β* = 0.24, respectively. The use of an orthographic spelling strategy was significantly predicted by pitch perception, *t*(95) = 2.64, *p* < 0.05; standardized *β* = 0.26, explaining 5.9% of the variance. Finally, tempo perception was a significant predictor of a morphological spelling strategy, *t*(95) = 2.01, *p* < 0.05; standardized *β* = 0.20, and explained 3.1% of the variance.

To summarize, using a stepwise regression procedure, we could identify various aspects of music processing abilities that contributed to the prediction of phonemic awareness and literacy skills: pitch perception predicted phonemic awareness and an alphabetic as well as orthographic spelling strategy. Tempo perception contributed to the prediction of reading comprehension and a morphological spelling strategy. Finally, rhythm reproduction was a predictor of phonemic awareness, the number of correctly spelled graphemes, as well as an alphabetic spelling strategy.

###### Unique contribution (over and above phonemic awareness) of music processing abilities to reading and spelling abilities in third-grade children

A stepwise regression procedure was chosen in which phonemic awareness was entered in the first block, and the five music processing measures were entered in the second block. With this approach, none of the music processing variables significantly predicted reading comprehension after the contribution of phonemic awareness was accounted for, while phonemic awareness explained 20.0% of the variance in reading comprehension (adjusted *R*^2^), *t* (95) = 4.97, *p* < 0.001; standardized *β* = 0.46. With respect to spelling, two models were significant for the prediction of the number of correctly spelled graphemes: In Model 1, phonemic awareness explained 22.9% of the variance, *t*(95) = 5.41, *p* < 0.001; standardized *β* = 0.49. In Model 2, phonemic awareness, *t*(95) = 4.24, *p* < 0.001; standardized *β* = 0.40, and rhythm reproduction, *t*(95) = 2.38, *p* < 0.05; standardized *β* = 0.23, explained together 26.6% of the variance in the number of correctly spelled graphemes. Concerning the use of an alphabetic spelling strategy, again two models were significant: In the first model, phonemic awareness was a significant predictor of an alphabetic spelling strategy, *t*(95) = 4.62, *p* < 0.001; standardized *β* = 0.43, explaining 17.6% of the variance. In the second model, a combination of phonemic awareness and rhythm reproduction explained 26.0% of the variance in the use of an alphabetic spelling strategy, *t*(95) = 3.23, *p* < 0.01; standardized *β* = 0.31, and *t*(95) = 3.41, *p* < 0.01; standardized *β* = 0.33, respectively (note that the two predictors make a comparably strong contribution here). Phonemic awareness was a significant predictor of an orthographic spelling strategy, *t*(95) = 4.16, *p* < 0.001; standardized *β* = 0.39, explaining 14.7% of the variance, while music processing abilities did not make a unique contribution to the use of that strategy. Finally, when phonemic awareness was entered first into the model, it was the only significant predictor of a morphological spelling strategy, explaining 8.2% of the variance, *t*(95) = 3.08, *p* < 0.01; standardized *β* = 0.30.

Taken together, these results indicate that music processing abilities do not make a unique contribution to the prediction of reading comprehension, over and above that of phonemic awareness. The same is true with respect to the prediction of an orthographic and morphological spelling strategy. After the predictive value of phonemic awareness is accounted for, music processing abilities do, however, still significantly contribute to the prediction of the number of correctly spelled graphemes and the use of an alphabetic spelling strategy: rhythm reproduction significantly predicted the number of correctly spelled graphemes as well as the use of an alphabetic spelling strategy. To the prediction of the latter, the contribution of rhythm reproduction was as strong as that of phonemic awareness.

## Discussion

In the two studies presented here (Study 1: preschool children; Study 2: third-graders), three tasks assessing temporal musical skills (tempo perception, rhythm perception, rhythm reproduction) and two tasks measuring tonal skills (pitch perception, contour perception), taken from two diagnostic instruments (*Musikscreening für Kinder I/Music Screening for Children I*, Jungbluth and Hafen, unpublished; MBEMA Peretz et al., 2013) and adapted for use with a tablet computer were used to assess relations between music processing and (a) phonological awareness (Study 1 and Study 2) and (b) reading and spelling (Study 2).

Of special interest for the study with preschool children (Study 1) was whether phonological awareness with respect to larger phonological units (termed *phonological awareness* in the following) would show a different pattern of relations to music processing abilities than phonological awareness with respect to smaller phonological units, i.e., phonemes (termed *phonemic awareness* in the following). Indeed, phonological awareness was significantly related to the two tonal tasks as well as to both rhythm processing tasks while phonemic awareness was related to the two rhythm processing tasks only. This result is in accordance with the findings of Degé et al. (2015), who also found more associations between phonological awareness and music processing abilities than between phonemic awareness and music processing abilities in German preschool children. What is more, these authors who used the *Music Screening for Children I* to assess musical abilities did report significant correlations between phonological awareness and (a) pitch perception, (b) rhythm perception, and (c) rhythm reproduction (although not for all phonological awareness subtests) and between phonemic awareness and rhythm reproduction (but not pitch perception and rhythm perception) abilities. Moreover, they also did not find significant relations between phonological/phonemic awareness and tempo perception as measured by the *Music Screening for Children I*. Thus, our results are largely consistent with those of Degé et al. (2015) even though these authors used a different diagnostic instrument to measure phonological and phonemic awareness (BISC; Jansen et al., 2002). Additionally, the results of both studies indicate that tonal skills as well as rhythm processing abilities are related to phonological awareness in preschool children, while only rhythm processing abilities are related to phonemic awareness in this age group.

Interestingly, in third-graders, phonemic awareness was related to rhythm processing skills as well as to the pitch perception measure. This corroborates former findings concerning relations between phonological/phonemic awareness and pitch processing (Lamb and Gregory, 1993; Loui et al., 2011) and pitch and rhythm processing (Anvari et al., 2002) in school-aged children. The results of our study concerning relations between rhythm processing abilities and phonemic awareness in third-graders in combination with the results from the study with preschool children indicate that rhythm processing abilities might be involved in the development of both phonological and phonemic awareness. They add to the growing body of evidence for links between musical rhythm processing and reading-related cognitive skills, such as phonological awareness (Ozernov-Palchik and Patel, 2018). Overall, the link between rhythm processing and phonological/phonemic awareness might be explained by shared mechanisms, such as the accurate processing of temporal patterns in acoustic stimuli (Moritz et al., 2013; Ozernov-Palchik et al., 2018). Temporal patterns in speech contain important cues to phonological units such as syllables, phonemes, and stresses (Ozernov-Palchik et al., 2018): With respect to larger phonological units, timing skills as indicated by rhythmical abilities might be among others relevant for the perception of stressed vs. unstressed syllables and for the accurate perception of speech rhythm (Corriveau et al., 2010). With respect to the phonemic level, efficient temporal processing abilities might be required for accurate phoneme perception, the quality of which determines the quality of the long-term representations of these phonemes and has an impact on children’s ability to learn grapheme–phoneme mappings (Manis et al., 1997). As phonemic awareness and reading ability seem to be reciprocally related to each other in the process of reading acquisition (Wimmer et al., 1991), this potential indirect influence of temporal processing on the ability to learn grapheme–phoneme mappings might in turn influence children’s phonemic awareness abilities.

In both studies presented here, the ability to process spectral information in music, i.e., tonal skills, was additionally related to phonological awareness (preschool children) and phonemic awareness (third-graders). The link between pitch perception and phonological/phonemic awareness might again be explained by shared mechanisms: on the phonemic level, the ability to process spectral information in the speech signal is relevant for phoneme perception as phonemes differ with respect to spectral/frequency content (e.g., Bohn and Polka, 2001). As pitch is the direct perceptual correlate of frequency, pitch perception and phoneme perception might share the same process of frequency discrimination (Loui et al., 2011). Regarding larger phonological units, pitch processing skills might be linked to phonological awareness as these skills are required to segment the speech stream (Ziegler et al., 2012).

Regression analyses revealed that in both age groups (preschool and Grade 3), rhythm reproduction was the strongest predictor of phonological and phonemic awareness, respectively. In preschool children, it explained about 17% of the variance in phonological awareness and 11% of the variance in phonemic awareness. In third-graders, rhythm reproduction explained about 13% of the variance in phonemic awareness. This consistent pattern underscores the special relevance of rhythm reproduction abilities for both phonological and phonemic awareness. The correlational analyses revealed for both age groups strong relations between rhythm perception and rhythm reproduction skills, which is not surprising, since the rhythm reproduction measure integrates perceptual and motor components. Of the two rhythm processing measures used in the current study, the reproduction measure prevailed in the regression analyses as the only significant predictor. This indicates that the association between phonological/phonemic awareness skills and a purely perceptual measure of rhythm processing is weaker than the association between phonological/phonemic awareness and a measure that combines perceptual and motor aspects of rhythm processing, such as the rhythm reproduction measure used in our study. Thus, phonemic and phonological awareness might depend not only on the perceptual processing of temporal cues in speech but also on motor and automatization skills. Indeed, studies with children have reported relations between the ability to clap in time and phonological awareness (Bonacina et al., 2018) and articulatory skills and phoneme awareness (Carroll et al., 2003; Thomas and Senechal, 2004). Jansen (1992) found that the recognition of consonants was influenced both by reafference processing of articulatory motor functions and acoustic processing itself. The relevance of motor, articulatory, and automatization skills for phonological development and the development of reading and spelling is also stressed in the cerebellar deficit hypothesis of developmental dyslexia (see, e.g., Nicolson et al., 2001). One might suspect that the relatively strong relations between rhythm reproduction and phonological/phonemic awareness are, at least in part, attributable to phonological short-term or working memory as the rhythm reproduction task requires storage of the perceived rhythms in short-term memory to be able to reproduce them correctly, while phonological/phonemic awareness tasks require both the storage and manipulation of words and phonological units. As a phonological short-term memory task and a phonological working memory task (digit span forward vs. backward) were included in Study 1 (preschool children), it was possible to test this assumption. Phonological *short-term memory* was related neither to rhythm reproduction nor to phonological and phonemic awareness, but phonological *working memory*, including a manipulation component, was related to phonological and phonemic awareness (but not to rhythm reproduction). Controlling for phonological working memory did not substantially reduce the strength of the relations between rhythm reproduction and phonological/phonemic awareness in preschool children. A second objection might be that the especially strong relations between rhythm reproduction and phonological/phonemic awareness stem from the fact that rhythm reproduction was measured in a more differentiated way (using a coding scheme for scoring the correctness of the reproduced rhythms) than the other music processing tasks. This possibility cannot be ruled out, but note the relatively high consistency between our results and those of Degé et al. (2015) described above: these authors used the same diagnostic instrument as we did to measure rhythm reproduction abilities (*Music Screening for Children I*) but analyzed the rhythm reproduction task using the standard and less differentiated way (maximal score = 20).

The second significant predictor in the regression analyses predicting phonological/phonemic awareness was pitch perception, explaining a significant proportion of the variance in phonological awareness (preschool children) and phonemic awareness (third-graders) in addition to rhythm reproduction abilities. This shows that temporal and tonal musical skills uniquely and distinctly contribute to the prediction of phonemic/phonological awareness. The question remains, however, why pitch perception turned out to be a significant predictor and correlate of phonemic awareness in third-graders but not in preschoolers. As mentioned above, Degé et al., 2015 using the same task from the *Music Screening for Children I*, also reported a lack of correlations between pitch perception and phonemic awareness tasks in German preschool children. As has already been stated, German preschoolers do not receive formal reading instruction. Thus, in contrast to school children, German preschool children cannot use their knowledge of grapheme–phoneme mappings as an aid in establishing phonemic awareness abilities. Instead, they need to base their phonemic awareness skills purely on their experience with the analysis and synthesis of phonemic units in spoken language. In doing so, they might more often make use of temporal than of spectral cues in the speech signal, which explains why their pitch processing abilities are not related to their phonemic processing abilities. However, this is purely speculative.

In third-graders, besides phonemic awareness skills, reading and spelling skills were measured, allowing us to investigate associations between music processing skills and literacy skills. Of special relevance was the contribution of music processing skills to the prediction of reading and spelling over and above that of phonemic awareness. As was expected, phonemic awareness was related to reading and spelling measures. When phonemic awareness was not used as a predictor in the regression analyses, the only significant predictor of reading skills was tempo perception, explaining about 3% of the variance in reading. When phonemic awareness was used as the first predictor in the regression analyses, music processing skills made no significant contribution to the prediction of reading skills. These results seem at odds with other studies reporting relations between reading and pitch perception (Lamb and Gregory, 1993; Anvari et al., 2002) or reading and rhythmic ability (Douglas and Willatts, 1994; Rautenberg, 2015). The lack of associations between rhythm processing abilities and reading might seem especially surprising as Huss et al. (2011) argued that rhythmic perception and production would be expected to affect the development of literacy in children across languages from different rhythm classes, and there is evidence that rhythm perception and production predict reading abilities in developmental dyslexia (Flaugnacco et al., 2014). It has to be kept in mind, however, that we used a reading comprehension test in the current study, testing the comprehension of words and sentences, to measure reading abilities in third-graders. It might be that a measure focused on more basic reading processes (synthetic reading, reading accuracy) would have better captured potential relations between music processing abilities and reading. Additionally, while many former studies focused on beginning readers (e.g., Lamb and Gregory, 1993; Anvari et al., 2002; Rautenberg, 2015; but see Douglas and Willatts, 1994), we studied children who had already received 2 years of reading instruction at school. Thus, these children possessed considerable reading expertise and it can be expected that they relied to a lesser extent on alphabetic/phonological reading strategies and to a larger extent on orthographic reading strategies (Frith, 1985, 1986) than less experienced readers, which might additionally explain the lack of relations between music processing and reading. Future studies should incorporate reading measures of basic reading abilities and include children in earlier phases of reading development.

With respect to spelling, music processing did not explain a unique amount of variance in the use of orthographic and morphological spelling strategies over and above that of phonemic awareness. This result was largely expected: orthographic and morphological spellings are based on larger phonological units and rely on orthographic rules and morphological knowledge. Thus, they should be less dependent on basic auditory processing abilities. Rhythm reproduction ability, however, did make a unique contribution to the prediction of alphabetic spelling and the number of correctly spelled graphemes. This is in accordance with findings of Overy (2003), who reported a relation between the skill of tapping out the rhythm of a song and spelling ability. How can these relations be explained? Alphabetic spelling relies on phoneme–grapheme mappings. Although German orthography exhibits a much higher regularity in the direction of graphemes to phonemes, i.e., for reading, than in the direction of phonemes to graphemes, i.e., for spelling (see Wimmer and Mayringer, 2002), it is quite common in Germany to base early spelling instruction on phoneme–grapheme mappings (Schründer-Lenzen, 2004; Füssenich and Löffler, 2005), thus promoting alphabetic spelling strategies. Rhythmic cues in language seem to facilitate the acquisition of phonological representations (Ozernov-Palchik and Patel, 2018), and the efficiency of temporal auditory processing affects phoneme perception and influences the quality of long-term representations of phonemes (Manis et al., 1997). This might affect children’s capability of learning these phoneme–grapheme mappings and might thus have an impact on alphabetic spelling abilities. The motor component involved in the rhythm reproduction task might be indicative of children’s ability to automatize skills and knowledge, which also influences spelling ability (Nicolson et al., 2001). Note, however, that the studies presented here were cross-sectional in nature and thus do not inform us about potential *causal influences* of rhythmic abilities on phonological awareness and literacy skills.

From an applied perspective, the results of the current study concerning relations between music processing skills and phonological/phonemic awareness as well as literacy skills lend support to the notion that the predictive validity of diagnostic tests measuring cognitive skills underlying literacy development might profit from the inclusion of non-linguistic tasks measuring the ability to process temporal and spectral information in music. Our results suggest that it might be fruitful to consider tonal and temporal musical skills here. Concerning temporal musical skills, rhythm reproduction showed the strongest associations with literacy- and phonological/phonemic awareness skills (phonological awareness in preschool children; phonemic awareness in both age groups; alphabetic spelling skills in third-graders after controlling for phonemic awareness). With respect to tonal skills, pitch perception showed the strongest, although much weaker, relations to these skills (to phonological awareness in preschool children and phonemic awareness in third-graders). As a pitch reproduction measure was not included into the study, the question remains whether it might be the combination of perceptual and motor components in a task which is especially predictive of literacy and phonological/phonemic awareness (see also Loui et al., 2011, who found relations between pitch processing and phonemic awareness only for a combined perception–production measure, not for a purely perceptual measure). Future research should include a stricter comparison of perception and reproduction measures. Additionally, more longitudinal studies are needed to address the question whether earlier musical abilities predict later literacy skills and whether musical skills contribute to their prediction over and above phonological processing skills. In a longitudinal study with school children by David et al. (2007), for example, rhythm reproduction correlated significantly with phonological awareness and naming speed (all measured in Grade 1) and with reading in Grades 1–5. But when phonological awareness was controlled for, rhythm reproduction was only a unique predictor of reading in Grade 5; when naming speed was controlled for, it was only a unique predictor of reading in Grades 2, 3, and 5. Finally, future studies should include larger sample sizes.

However, with respect to the potential diagnostic value of musical tasks for the prediction of reading-related skills, methodological issues also need to be addressed. In the preparation of our study, we faced the challenge of selecting an assessment for children between 5 and 8 years of age that includes the potentially relevant variety of music perception skills, has satisfying psychometrical quality and is adequate for group testing. Of course, there is a long tradition – especially in music psychology and music education – of assessing musical abilities, and a corresponding amount of test instruments is available (for reviews, see, e.g., Boyle and Radocy, 1987; Law and Zentner, 2012; Schellenberg and Weiss, 2013; Müllensiefen and Hemming, 2018). However, regarding the focus of our research, it is worth mentioning that there are only very few test instruments for preschool and primary school children (e.g., Gordon, 1979, 1989). Furthermore, most of these instruments cover only a very limited area of music perception skills (e.g., Gordon, 1979, 1989), they do not match standard psychometric criteria (Platz et al., 2015), or they do not provide an adequate documentation. Another problem is that most assessments were developed between 1960 and 1990 (e.g., Bentley, 1966; Wing, 1968; Gordon, 1989) and thus do not meet the state of the art of stimulus sound production and computer-based test distribution (Law and Zentner, 2012; Hasselhorn and Knigge, in press) or, as Schellenberg and Weiss (2013) put it: “Unfortunately, there is no test of music aptitude that is considered to be the ‘gold standard’.”

Hence, we decided to use two assessments that have been widely and successfully used in several studies (e.g., Bastian, 2000; Clément et al., 2015; Degé et al., 2015; Rautenberg, 2015; Soleimanifar et al., 2016) that deal with music perception areas relevant to our research interest and that include an age range as close as possible to our target group: namely, the *Music Screening for Children I* (Jungbluth and Hafen, unpublished) and the MBEMA (Peretz et al., 2013). There were, however, relevant limitations of these test instruments regarding our scope of application: namely, they were published as paper-and-pencil tests and are intended for individual testing (*MBEMA*) or use a suboptimal setup for group testing (*Music Screening for Children I*). Furthermore, detailed psychometric data have either not been published (*Music Screening for Children I*) or are rather sparse to date (*MBEMA*).

Against this background, we first performed a transformation of the tests into a computer-based assessment and, second, conducted piloting and psychometric evaluation regarding the two adapted versions (see Knigge et al., unpublished). Item selection process and reliability measures confirmed the procedure as necessary. Besides securing reliability of the tests, the procedure yielded shortened sub-tests that allow for shorter testing time – especially important for children ages 5–8 – while keeping assessment accuracy high. Thus, at the methodological level, our study also aims to contribute to the development of reliable and economically usable test instruments in the area of music perception (see Knigge et al., unpublished).

If music processing abilities were indeed predictive of phonological and literacy skills, a training of musical skills might also show a transfer to these skills. Indeed, a number of recent studies have been promising in this respect showing effects of musical training on phonological awareness (Degé and Schwarzer, 2011; Moritz et al., 2013) or reading skills (Moreno et al., 2009; Rautenberg, 2015; Hallam, 2018) or both (Flaugnacco et al., 2015) or on phonological skills and spelling (Overy, 2003). For future research concerning the promotion of literacy skills and literacy-related skills, a consecutive training regime combining musical training (helping to enhance the processing of temporal and spectral auditory cues in speech and thus supporting the development of phoneme perception and phonological processing) with training in phonological awareness might be an especially fruitful approach. However, a recent training study by Kempert et al. (2016) did not find incremental training effects on phonological awareness in an intervention group receiving music training prior to a phonological awareness intervention in comparison to a second intervention group receiving a training of phonological awareness only. This lack of effects might be attributable to low training intensity as well as the fact that a broad range of music facets was trained (Kempert et al., 2016). Concentrating on the training of specific music processing skills such as rhythm processing or other temporal musical skills might be more promising (see, e.g., Overy, 2003; Moritz et al., 2013; Hallam, 2018). In this respect, results from basic research on relations between music processing skills and phonological/literacy skills such as those we reported here can inform and inspire applied research dealing with diagnostic and training approaches in the context of cognitive skills relevant for the development of reading and spelling.

## Ethics Statement

The study was carried out in accordance with relevant Thuringian laws (Thuringian school law, Thuringian data protection law) and followed the recommendations of the Thuringian Ministry of Education, Youth, and Sports. Following the guidelines for conducting empirical studies in Thuringian schools, the protocol was approved by the local school authorities (Staatliches Schulamt Mittelthüringen). The children’s parents gave written informed consent in accordance with the Declaration of Helsinki.

## Author Contributions

CS, JK, GM, and SS contributed to conception and design of the studies. JK was responsible for the procedure for adapting the music tasks into a computer-based assessment. CS, GM, SS, and AW organized data collection and data handling. CS, JK, and GM performed the statistical analysis. CS wrote the first draft of the manuscript. JK, GM, and SS wrote sections of the manuscript. All authors contributed to manuscript revision.

## Conflict of Interest Statement

The authors declare that the research was conducted in the absence of any commercial or financial relationships that could be construed as a potential conflict of interest.
